# Analyzing Prognostic Hub Genes in the Microenvironment of Cutaneous Melanoma by Computer Integrated Bioinformatics

**DOI:** 10.1155/2022/4493347

**Published:** 2022-03-08

**Authors:** Guangyao Li, Jingye Zhang, Yourao Liu, Xiqing Cheng, Kai Sun, Wenjuan Hong, Ke Sha

**Affiliations:** ^1^Affiliated Hospital of Jiujiang University, No. 57 Xunyang East Road, Jiujiang 332000, Jiangxi Province, China; ^2^First Affiliated Hospital of Guangxi Medical University, Nanning 530021, Guangxi Zhuang Autonomous Region, China

## Abstract

Cutaneous melanoma (CM) is attracting increasing attention due to high mortality. In response to this, we synthetically analyze the CM dataset from the TCGA database and explore microenvironment-related genes that effectively predict patient prognosis. Immune/stromal scores of cases are calculated using the ESTIMATE algorithm and are significantly associated with overall patient survival. Then, differentially expressed genes are identified by comparing the immune score and stromal score, also prognostic genes are subsequently screened. Functional analysis shows that these genes are enriched in different activities of immune system. Moreover, 19 prognosis-related hub genes are extracted from the protein-protein interaction network, of which four unreported genes (IL7R, FLT3, C1QC, and HLA-DRB5) are chosen for validation. A significant negative relationship is found between the expression levels of the 4 genes and pathological stages, notably *T* grade. Furthermore, the K-M plots and TIMER results show that these genes have favorable value for CM prognosis. In conclusion, these results give a novel insight into CM and identify IL7R, FLT3, C1QC, and HLA-DRB5 as crucial roles for the diagnosis and treatment of CM.

## 1. Introduction

Cutaneous melanoma (CM) is highly malignant, accounting for more than 80% of deaths from skin disease [1, 2]. As of late, the incidence and mortality of CM are increasing. Fortunately, innovative immunotherapy strategies, including therapies using anti‐PD1, anti‐PD-L1, and anti‐CTLA4, have effectively improved patient prognosis [3–5]. However, only approximately 40% of patients who receive immunotherapy are able to achieve effective long-term remission [6]. Besides, some patients experience adverse effects due to the complicated interaction between the tumor microenvironment and cancer cells [7]. Therefore, identifying more effective prognostic biomarkers is of extraordinary importance for the therapy of CM.

The tumor microenvironment is composed of immune cells and stromal cells, which is of great significance for tumor diagnosis and prognostic assessment [8, 9]. High levels of immune cell infiltration can be regarded as an indicator of a favorable prognosis in cancers [10], implying that effectively assessing the condition of the microenvironment might hold promise for further treatment. In addition, techniques for anticipating tumor purity utilizing gene expression information from The Cancer Genome Atlas (TCGA) database have been created [11, 12]. For instance, Yoshihara et al. [11] designed an algorithm called ESTIMATE, which has been used to analyze cancer data in numerous studies, showing the efficiency of the algorithm [13–15]. As a frontier study, we want to explore the microenvironment-related genes that effectively predict CM development by integrating the TCGA database and immune-related scores.

## 2. Materials and Methods

### 2.1. Data Acquisition and Grouping

The data about gene expression of CM patients was obtained from the TCGA database (https://portal.gdc.cancer.gov/), which offers the quantified expression levels of mRNA in the form of fragments per kilobase per million (FPKM). Dataset was submitted by the Genomic Data Commons using the Illumina platform (August 15, 2017). The corresponding clinical information included gender, age, TNM grade, and New-American Joint Committee on Cancer (AJCC) grade; data on survival and prognosis were also acquired from the TCGA database. Moreover, information on identified gene alterations was obtained from the cBioPortal database (https://www.cbioportal.org/). Immune scores and stromal scores were determined with the ESTIMATE algorithm [11] on the downloaded data. Because no other valid CM datasets could be found in other public databases, 470 patients were randomly divided into an experimental group and a testing group for analysis and validation.

### 2.2. Differential Analysis of Expressed Genes

The data processing was carried out using the ‘limma' package [16] in R software. The thresholds for definite differentially expressed genes (DEGs) were set to adj. *p* < 0.05 and fold change >2.

### 2.3. Heatmaps and Clustering Analysis

Heatmaps and clustering of DEGs were created utilizing the ‘heatmap' (R package). Correlation heatmaps of the identified genes were drawn with the ‘corrplot' (R package). The Pearson correlation coefficient was used for correlation analyses, in which >0 shows a positive relationship and >0.5 shows a strong relationship.

### 2.4. Visualization of Gene Expression Levels and Chromosome Locations

The R package ‘OmicCircos' [17] was utilized to visualize the expression levels and chromosome areas of the top 100 significant DEGs.

### 2.5. Functional Analysis of DEGs

Functional enrichment analysis of the DEGs utilizing the David online dataset [18] was performed to identify Kyoto Encyclopedia of Genes and Genomes (KEGG, https://www.genome.jp/kegg/) pathway and GO categories, the later including biological processes (BPs), molecular functions (MFs), and cellular components (CCs). A false discovery rate (FDR) <0.05 was used as cut-off criterion. The outcome of the enrichment analyses was pictured by the ‘GOplot' package [19].

### 2.6. Clinical Correlation and Survival Analysis of Genes

The R package ‘ggstatsplot' (https://cran.rproject.org/web/bundles/ggstatsplot/) was used to reveal the relationships between the gene expression levels and patient clinical features. Independent samples *T* test or one-way analysis of variance (ANOVA) was used as fitting. The survival analysis was led utilizing the ‘survial' (https://CRAN.R-project.org/package=survival) package to evaluate the correlation between the overall survival (OS) and the gene expression level; the K-M curves were drawn using the ‘survminer' (https://CRAN.Rproject.org/package=survminer) package. The correlation was determined by the log-rank test, with *p* < 0.05 as the threshold.

### 2.7. Protein-Protein Interaction Analysis

A protein-protein interaction (PPI) network was constructed to demonstrate the association among the proteins encoded by the identified DEGs. The network was created through STRING version 10.5 (https://string-db.org/), and the outcomes with a minimum correlation score of 0.4 were reconstructed via Cytoscape programming [20]. The connectivity degree of each node in the organization was also calculated. In addition, cytoHubba, a Cytoscape module application that provides an easy way to analysis significant hubs in biological networks [21], was used for comprehensive evaluation.

### 2.8. TIMER Online Analysis of Identified Genes

To analyze the relationship of the expression level of the identified genes with tumor purity and the infiltration of immune cells (B cells, CD4+ T cells, CD8+ T cells, neutrophils, macrophages, and dendritic cells), we used the web-based instrument TIMER (https://cistrome.shinyapps.io/clock/) [22].

## 3. Results

### 3.1. Immune/Stromal Scores Are Both Significantly Associated with Different Pathological Types

**Figure 1:**
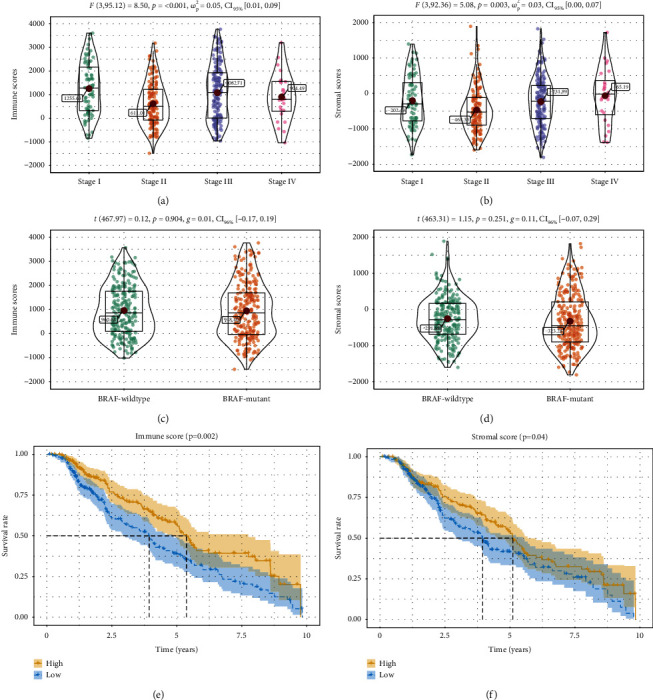
Immune scores and stromal scores are associated with CM pathological types and their overall survival. (a) Distribution of immune scores of CM pathological types. Violin plot shows that there is significant association between CM pathological types and immune scores (*p* < 0.001). (b) Distribution of stromal scores of CM pathological types. Violin plot shows that there is significant association between CM pathological types and stromal scores (*p*=0.003). (c) Distribution of immune scores for BRAF mutant and BRAF wildtype CM cases. Violin plot shows that there is no significant association between BRAF mutation status and immune scores (*p*=0.904). (d) Distribution of stromal scores for BRAF mutant and BRAF wildtype CM cases. Violin plot shows that there is no significant association between BRAF mutation status and stromal scores (*p*=0.251). (e) CM cases were divided into two groups based on their immune scores: the top half of 235 cases with higher immune scores and the bottom half of 235 cases with lower immune scores. As shown in the Kaplan–Meier survival curve, median survival of the high score group is longer than low-score group (*p*=0.002). (f) Similarly, CM cases were divided into two groups based on their stromal scores: the top half of 235 cases and the bottom half of 235 cases. The median survival of the high score group is longer than the low-score group (*p*=0.04).

We obtained the gene data and clinical profiles of 470 CM patients from the TCGA database. Among these patients, 180 (38.3%) were female, and 290 (61.7%) were male. The average age of the patients was 58.2 years old, and 45.1% were over 60 years of age. All CM patients with complete gene data and clinical data in the TCGA database were included for this study. Through the ESTIMATE algorithm, immune scores went from −1,481.02 to 3,768.83, and stromal scores were distributed between −886.07 and 584.93 (Figures 1(a) and 1(b)). The average immune score of stage I patients was the highest among the four grades, and the average scores of stage II and stage IV patients were the two least. Differences in immune scores among the four stages were statistically significant (Figure 1(a), *p* < 0.001). Interestingly, the order of the stromal scores of the different stages from highest to lowest, is as follows: stage IV > stage III > stage I > stage II (Figure 1(b), *p* < 0.01), showing that immune scores might be more significant in the relationship of pathological types than stromal scores, despite they all had obvious differences between various stages.

In light of the AJCC melanoma guideline, BRAF mutations are frequent in CM patients and play a significant part in the development of CM [23]. We mapped the immune/stromal scores dependent on the BRAF mutation status in CM patients. Cases with BRAF mutation had lower immune scores and stromal scores, though this distinction was not measurably huge (Figures 1(c) and 1(d)).

To investigate the relationship between OS and the immune scores and/or stromal scores, we divided the 470 patients into high- and low-score groups as indicated by their scores. As shown in the Kaplan-Meier (K-M) survival analysis (Figure 1(e)), the middle OS of patients with a high immune score was higher than that of patients with a low score (*p*=0.002). Consistently, patients in the high score group of stromal scores had a longer middle OS than those in the low-score group (Figure 1(f), *p*=0.04).

### 3.2. Differential Analysis of Expressed Genes with Immune/Stromal Scores

**Figure 2:**
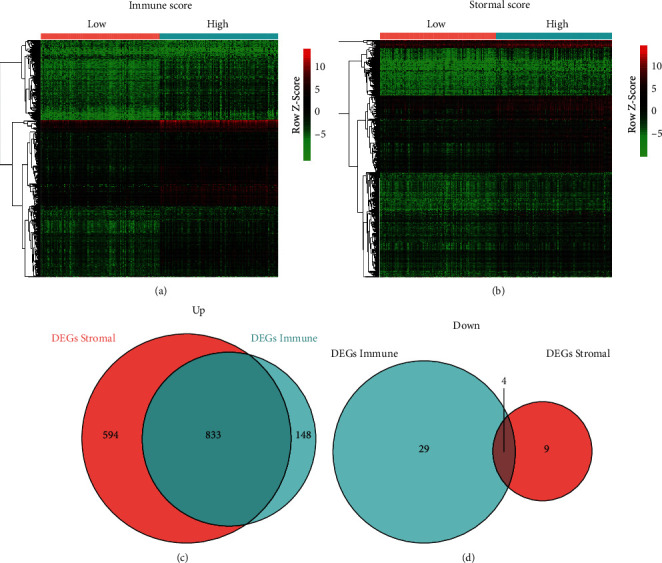
Differential analysis of expressed genes with immune/stromal scores in CM. Heatmaps were drawn based on the average linkage method and Pearson distance measurement method. Genes with higher expression are shown in red, genes with lower expression are shown in green, and genes with same expression level are in black. (a) Heatmap of the DEGs of immune scores of top half (high score) vs. bottom half (low score). (b) Heatmap of the DEGs of stromal scores of top half (high score) vs. bottom half (low score). Venn diagrams showing the number of commonly upregulated (c) or downregulated (d) DEGs in stromal and immune score groups.

To explore the DEGs related to the immune scores and/or stromal scores in CM, we analyzed each of the 235 cases in the experimental group. Immune-related genes were screened by contrasting the high score group and the low-score group; 981 genes were upregulated and 94 genes were downregulated in the gathering with high immune scores. A comparison of the stromal scores show that 1,427 genes were upregulated and 28 genes were downregulated in the high score group (Figures 2(a) and 2(b)). Moreover, there were 833 co-upregulated genes and 4 co-downregulated genes in both the high immune and stromal groups (Figures 2(c) and 2(d)). To get more accurate results, we focused on 837 common DEGs for subsequent analysis in this study.

### 3.3. Visualization of Gene Expression Levels and Chromosome Locations

**Figure 3:**
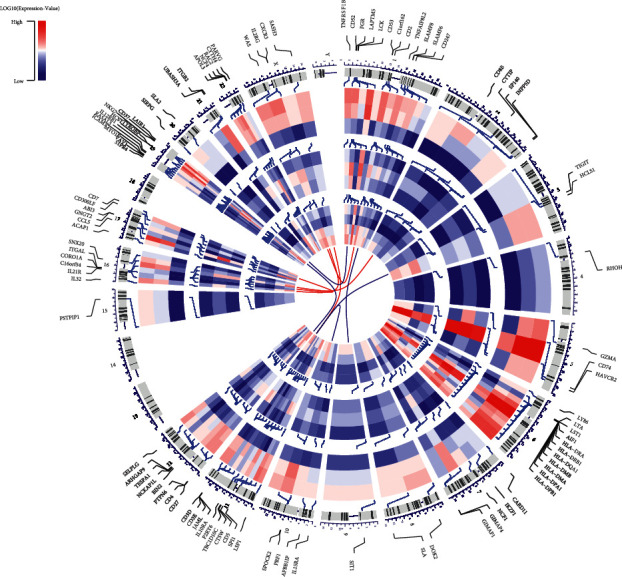
Circular visualization of connectivity, expression levels, and chromosomal positions of top 100 significant DEGs. The 10 CM cases randomly selected from 235 patients based on survival time (1–10 years), and their datasets were shown in the inner circular heatmaps (from inside to outside). Red represents high level gene expression, blue indicates low level of gene expression. The outer circle indicates chromosomes; lines coming from each gene point to their specific chromosomal locations. The top 5 genes according to adj. *p* or FC value are shown in red and blue, respectively, and connected with red and blue lines in the center of circles.

The gene levels and chromosomal locations of the top 100 significant DEGs (Figure 3) were visualized in 10 randomly chose cases from the experimental group based on survival time (1–10 years). These DEGs were distributed in chromosomes other than chromosomes 13/14/18/Y, and chromosomes 3/6 contained the most genes. Besides, the top 5 genes as indicated by the adj. *p*, i.e., SASH3, GNGT2, SNX20, WAS, and CORO1A, were situated in *X*, 17, 16, *X*, and 16, separately. The top 5 genes (CD8B, SIRPG, NKG7, PRF1, and CD3E) according to the fold change were distributed on chromosomes 2, 20, 19, 10, and 11, respectively.

### 3.4. Functional Enrichment and Survival Analysis of DEGs

**Figure 4:**
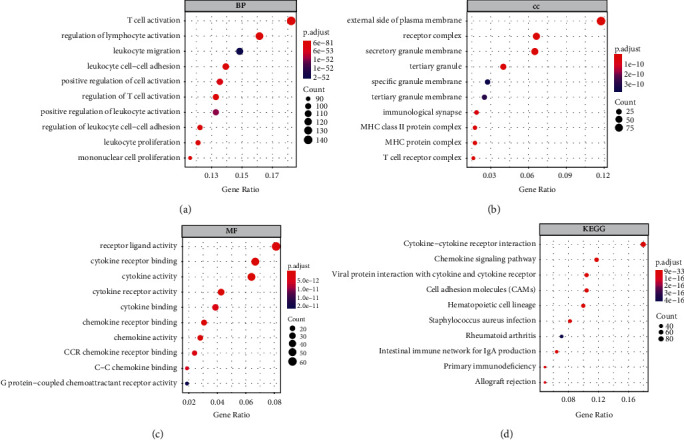
Functional annotation of the common DEGs. (a) Biological process GO term for the common DEGs. (b) Cellular component GO term for the common DEGs. (c) Molecular function GO term for the common DEGs. (d) KEGG pathway analysis for the common DEGs.

**Figure 5:**
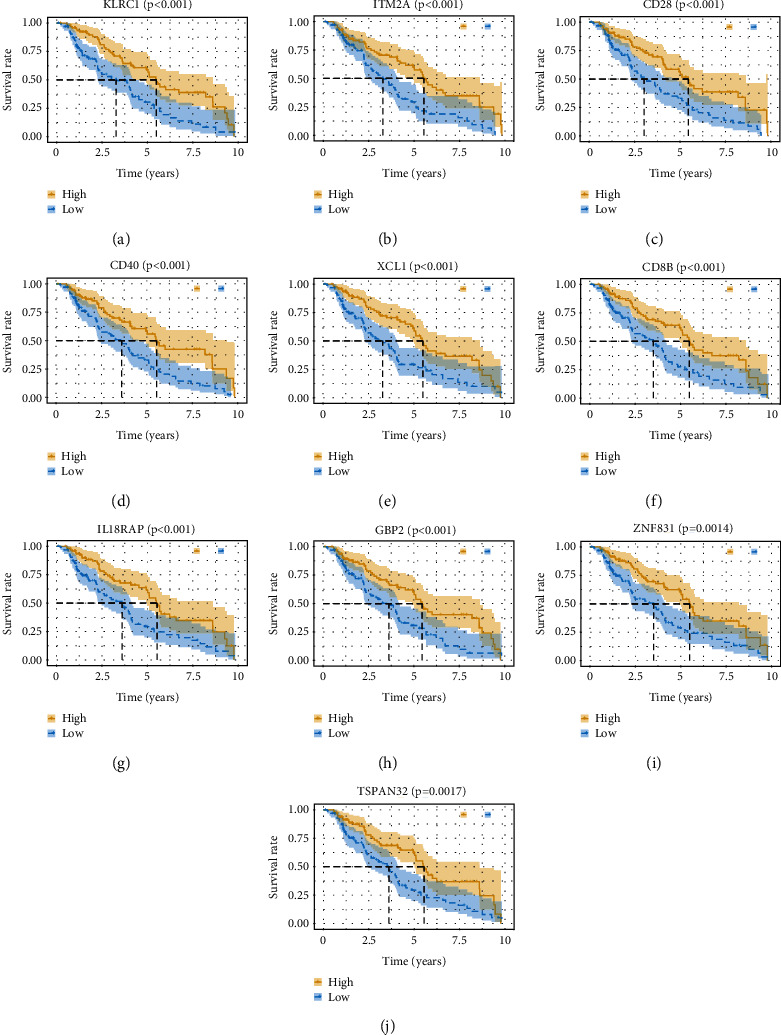
Association between expression level of individual DEGs and overall survival. Kaplan–Meier survival curves (a–j) were plotted for top 10 significant DEGs (KLRC1, ITM2A, CD28, XCL1, CD8B, IL18RAP, ZNF831, and TSPAN32) extracted from the comparison of groups of high (yellow line) and low (blue line) gene expression, *p* < 0.05 in log‐rank test.

To reveal the potential function of 837 DEGs, GO and KEGG pathway investigations were conducted. The most significant GO terms for BPs, MFs, CCs, and KEGG pathways are shown in Figure 4. To investigate the value of each DEGs in patient prognosis, we plotted K-M survival curves based on the patient data. Within 837 DEGs, a sum of 277 genes were demonstrated to be significantly correlated with prognosis in the log-rank test (*p* < 0.05); the top 10 significant genes are shown in Figure 5.

### 3.5. Functional Analysis of Prognostic DEGs

**Figure 6:**
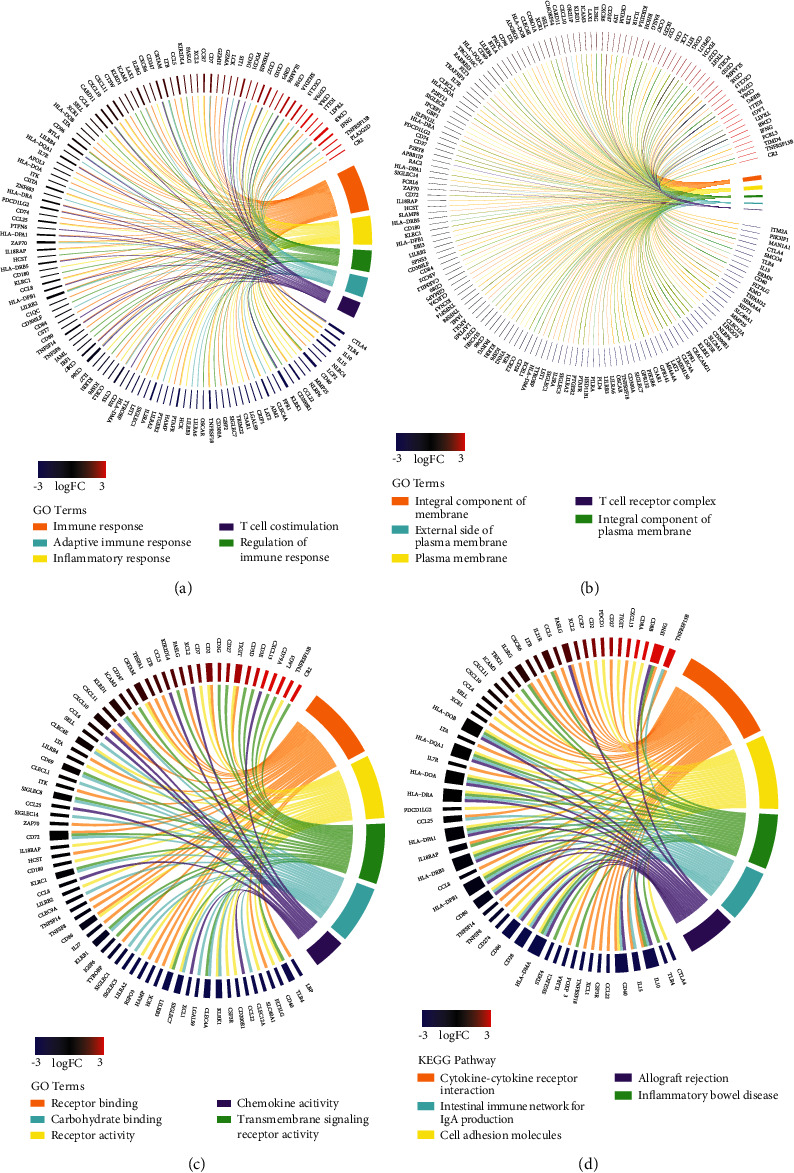
GO and KEGG analysis of prognostic DEGs. (a) Chord plot depicting the relationship between identified genes and GO terms of biological process. (b) Chord plot depicting the relationship between identified genes and GO terms of cellular component. (c) Chord plot depicting the relationship between identified genes and GO terms of molecular function. (d) Chord plot depicting the relationship between genes and KEGG pathways.

To better comprehend the function of the prognostic DEGs, which were chosen for further functional analysis. We found that their function was enriched in various GO terms, like the immune response, inflammatory response, T cell costimulation, the regulation of the immune response and the adaptive immune response (Figure 6(a)). As far as cellular components, the external side of the plasma membrane was the top GO term (Figure 6(b)). In addition, some molecular component GO terms, like receptor activity and transmembrane signaling receptor activity, were enriched (Figure 6(c)). Through the KEGG pathway, we found the cytokine-cytokine receptor interaction was highly associated with these genes (Figure 6(d)).

### 3.6. Protein-Protein Interactions and Correlation Analysis of Genes

**Figure 7:**
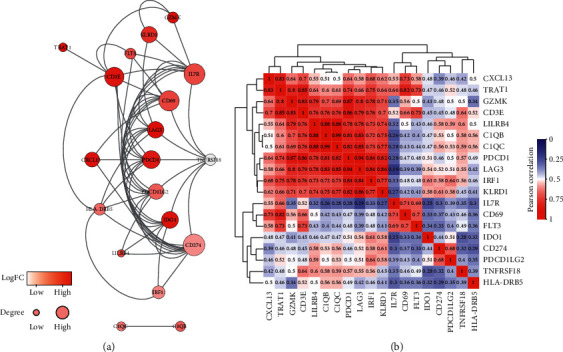
PPI network and clustered heatmap of 19 hub genes. (a) The color of the node in the PPI network reflects the log(FC) value of the Z score of gene expression, and the size of node indicates the number of interacting proteins with the designated protein, and the thickness of the edges represents the connectivity degree. (b) A clustered heatmap of Pearson correlation coefficients over 19 hub genes. Highly significant correlations are marked in red, significant ones in white, and low ones in blue.

To elaborate the interplay among the proteins encoded by the 277 prognostic DEGs, we utilized the STRING program to construct a PPI network, which included 237 nodes and 2,676 edges. Additionally, 40 of the 277 DEGs were excluded from the PPI network because their interaction scores were lower than 0.4. Among the 237 genes in the network, 19 hub genes (CD3E, CD69, CD274, KLRD1, PDCD1, LAG3, HLA-DRB5, IL7R, LILRB4, TNFRSF18, TRAT1, PDCD1LG2, C1QB, GZMK, C1QC, CXCL13, FLT3, IDO1, and IRF1) were screened according to the comprehensive assessment with 12 algorithms in the cytoHubba plugin, and there were strong positive correlations between these genes (Figures 7(a) and 7(b)). We also obtained more information about their alterations through cBioPortal (https://www.cbioportal.org/), as shown in Table 1. Moreover, four (IL7R, FLT3, C1QC, and HLA-DRB5) of the 19 genes were selected for subsequent analysis because they have not been recently reported to be correlated with clinical outcome of CM.

### 3.7. Correlation of the Four Hub Genes' Expression with Clinical Features

**Figure 8:**
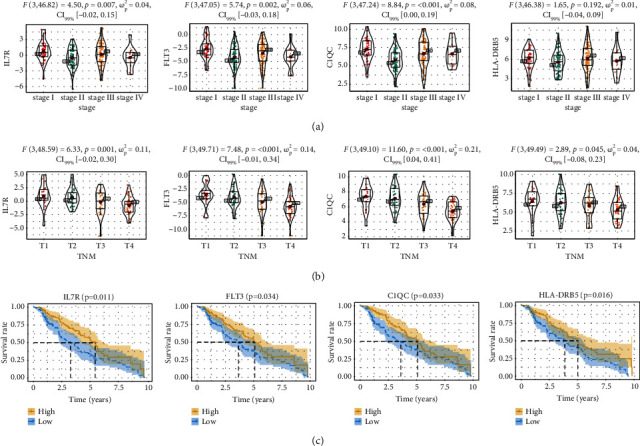
The 4 hub genes are associated with pathological types and overall survival in CM. (a) IL7R, FLT3, C1QC, and HLA-DRB5 genes differently express between different pathological types. (b) Expression of IL7R, FLT3, C1QC, and HLA-DRB5 in CM cases with different T stages. (c) Association between IL7R, FLT3, C1QC, and HLA-DRB5 expression and overall survival in CM. The yellow line indicates the samples with highly expressed genes (above the median-expression value), and the blue line indicates the samples with lowly expressed gene (below median-expression value).

To further reveal the value of the 4 hub genes, we explored the correlations between their expression levels and pathological types as well as OS. All 4 hub genes were essentially differentially expressed in CM patients with various *T* grades, although three (IL7R, FLT3, and C1QC) of them showed significant differences between pathological types, with lower expression levels indicating advanced pathological degrees and higher *T* grades (Figures 8(a) and 8(b)). In addition, Figure 8(c) showed that a high expression level of four genes is favorable for patient prognosis. The above results demonstrated that the 4 hub genes have great clinical value for CM.

### 3.8. Correlation of the Four Hub Genes' Expression with the Tumor Microenvironment

**Figure 9:**
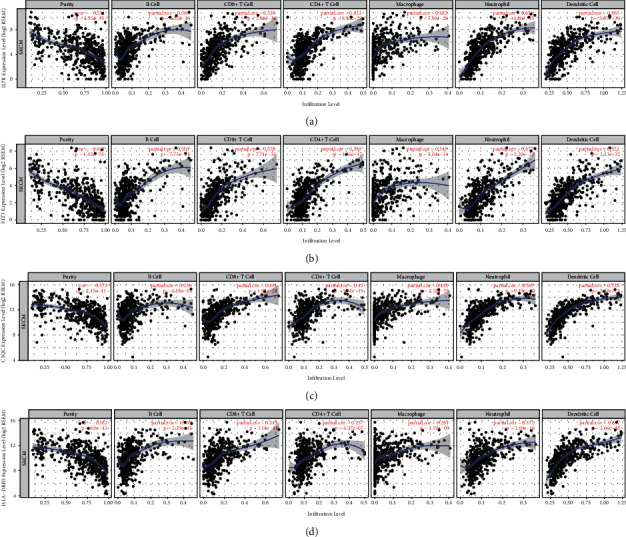
Association of 4 hub genes expression with immune infiltration in CM. (a) IL7R. (b) FLT3. (c) C1QC. (d) HLA-DRB5. *P* < 0.05 denotes significance. Each dot represents a sample in the TCGA-CM dataset.

The tumor microenvironment comprises of cancer cells, infiltrating immune cells, and stromal cells. We used TIMER to reveal relationships between the expression levels of the 4 genes and both the proportion of immune cells and tumor purity. Remarkably, IL7R, FLT3, C1QC, and HLA-DRB5 were all negatively correlated with cancer purity. Similarly, higher expression levels of the 4 genes obviously associated with a high infiltration of B cells, CD4+ T cells, CD8+ T cells, neutrophils, macrophages, and dendritic cells (Figures 9(a)–9(d)).

### 3.9. Validation of the Four Hub Genes in the Testing Group

**Figure 10:**
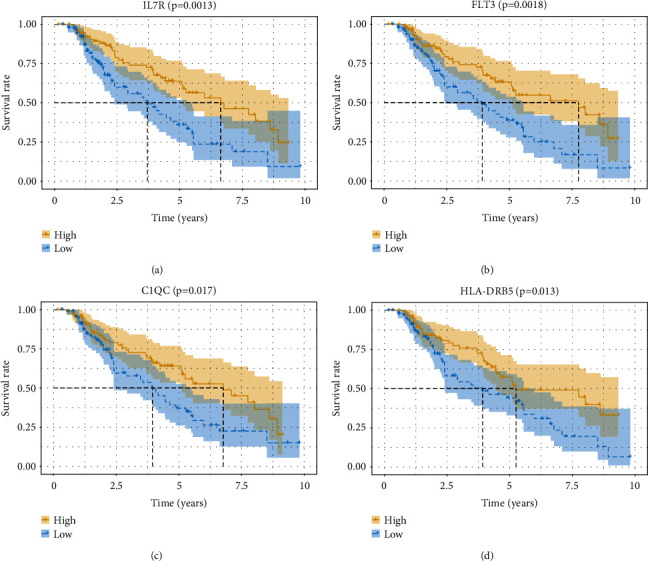
Validation of 4 hub genes in the testing group. (a–d) Association between IL7R, FLT3, C1QC, and HLA-DRB5 expression and overall survival in the testing group. The yellow line indicates samples with highly expressed genes (above the median-expression value), and the blue line designates the samples with lowly expressed genes (below median-expression value).

To verify whether the 4 hub genes identified from the experimental group also have prognostic value in the testing group, we plotted K-M survival curves of each gene based on the data of the testing group. Notably, each of the 4 hub genes was approved to be essentially linked to the clinical outcome of CM patients (Figure 10).

## 4. Discussion

CM is one of the most aggressive tumors, which has drawn increasing attention worldwide. Although some advanced treatments have been used in clinical practice, the results are still unsatisfactory [5]. Therefore, it is urgent to look for better biological biomarkers that could be used to guide clinical treatment. Melssen et al. [24] have demonstrated the significant correlation between the tumor microenvironment and melanoma, so we hope to discover new biomarkers that are related with patient prognosis by analyzing CM data from the TCGA database. Interestingly, through comparing expression level of genes in the CM dataset with high versus low immune/stromal scores, 277 prognostic genes were implicated in T cell activation and receptor ligand activity. Besides, 4 of the 19 hub genes have not recently been reported and were validated in the testing group.

To start with, we got 837 DEGs through a differential analysis between the parts of high and low immune/stromal score, and most of the DEGs were selected to participate in immune-related processes in the cancer microenvironment, as shown by the functional enrichment analysis (Figure 3). The results indicated that the interaction of immune cells and extracellular matrix molecules is critical for constructing the tumor microenvironment of CM, which is steady with past reports [25–27]. Figure 3 shows a positive association between expression level and prognosis of CM, and most genes were located on chromosomes 6/19. References [28, 29] demonstrated that the change of chromosome 6 is the most frequent karyotypic abnormality in melanoma, which is significantly associated with primary tumor development.

Then, 277 prognostic genes were screened through the OS analysis of 837 DEGs in CM patients. A PPI network was constructed with the identified genes that were significantly associated with the immune/inflammation response. By constructing the network, 19 highly interconnected genes were identified, of which 15 genes (e.g., CD3E, CD69, CD274, KLRD1, PDCD1, LAG3, LILRB4, TNFRSF18, TRAT1, PDCD1LG2, C1QB, GZMK, CXCL13, IDO1, and IRF1) were shown to be correlated with the outcome of CM patients in past reports [30–32], further indicating that our results based on big data mining are valid. The other four genes not previously discovered to be correlated with the outcome of CM may likewise be crucial for the development of tumor, which include the interleukin receptor encoding gene IL7R, cytokine receptor FLT3, complement component C1QC, histocompatibility complex HLA-DRB5. Through the analysis of the correlations between these four genes and clinical features, we observed a critical negative relationship among gene expression levels with pathological stages, particularly *T* grade. Furthermore, the TIMER results for each identified gene showed that all of them were negatively associated with tumor purity, while their expression levels were positively correlated with the infiltration of immune cells in CM (Figures 9(a)–9(d)).

Finally, by cross validation with the testing group, a valid dataset of 235 CM cases, the expression of the IL7R, FLT3, C1QC, and HLA-DRB5 genes was significantly correlated with patient prognosis (Figure 10). In previous report, IL7R has been distinguished as an important part in the development of immune cells, like lymphocytes [33]. Chandran et al. [34] observed that selectively expressed IL7R of *T* effector clones could significantly give rise to long-live memory cells after receptive exchange to cancer patients and considered IL7R as a vital part in the immune system of tumor. FLT3 propagates signals in the cell to maintain cellular functional activity, and its ligand is also important for inducing cancer relapse and antitumor immune reactions [35]. Powell et al. [36] reported that FLT3 could contribute on the penetration of CD8+ T cells and stimulatory dendritic cells in the microenvironment, further affecting the growth of cancers. C1QC, a crucial part of the classical complement pathway, yet there are few studies on C1QC in CM. Therefore, we hope to explore the biological value of C1QC through C1QB, basing on the high relationship between them (Figure 7(b)). Recent studies announced C1QB as a strong biomarker for the classification of CM patients, which could be utilized for both early discovery of melanoma and follow-up monitoring of patients.[37, 38]. Also, the protein encoded by HLA-DRB5 constitutes the HLA class II molecule, which assumes a focal part in the activity of immune cells [39]. Bierer et al. [40] found that the HLA class II genotype can effectively predict the reaction of renal cancer to combined immunochemotherapy and achieve a better prognosis of patients. Other studies also proposed that HLA class II expression can be seen as a good prognostic factor for malignant, while the lack of its expression might lead to bad outcome of patients [41]. These reports further confirm the validity of our results.

The interaction between CM and its tumor microenvironment significantly influences cancer development and further affects its diagnosis, treatment and prognosis. Previous studies have provided much evidence on how the activity of cancer characteristic genes contributes to the status of the tumor microenvironment. Within our research, we focused on the features of microenvironment-related genes that can influence patient prognosis by affecting the development of CM. Critically, our findings could provide effective data for elaborating the intricate interaction between the CM and its microenvironment.

## 5. Conclusion

In this research, we discovered 19 hub genes closely related to CM prognosis, of which 4 genes (IL7R, FLT3, C1QC, and HLA-DRB5) unreported were validated to be significantly favorable for patient outcome in the CM testing dataset and may become crucial biomarkers for CM. Furthermore, it would be fascinating to investigate whether this new group of genes could bring more prominent prognostic value than a single gene. Thus, more studies are needed to explore the potential association between CM prognosis and tumor microenvironment in a more comprehensive way.

## Figures and Tables

**Table 1:** The type and frequency of 19 hub gene alterations in CM (cBioPortal).

Categories	Gene symbol	Mutation	Amplification	Homozygous deletion	Up regulation	Multiple alterations	Total alteration
Cell surface	CD3E	0.27	0	2.97	5.41	0	8.65
	CD69	1.08	1.08	0.27	2.97	0	5.4
	CD274	0.27	1.08	1.35	2.16	0.27	5.13
	KLRD1	1.62	1.08	0.27	5.14	0	8.11
	PDCD1	1.08	0	2.16	5.14	0	8.38
	LAG3	1.36	0.81	0.27	5.15	0	7.59
	**IL7R**	7.84	3.24	0	3.24	0.54	14.86
	**HLA-DRB5**	0.27	0	0	4.32	0	4.59
Toll-like receptors	LILRB4	9.19	0.27	0	5.95	0.54	15.95
	TNFRSF18	1.08	1.08	1.35	4.86	0	8.37
	TRAT1	3.78	0.27	0	6.49	0	10.54
	PDCD1LG2	0.54	0.81	1.35	3.78	0.54	7.02
Complements	C1QB	1.89	1.62	0.81	6.49	0	10.81
	GZMK	1.08	0.54	0	4.59	0.27	6.48
	**C1QC**	2.16	1.35	0.81	6.49	0.54	11.35
Integral component of membrane	CXCL13	0.54	0.27	0	3.24	0	4.05
	**FLT3**	8.65	0.27	0	4.86	0	13.78
Other	IDO1	2.97	0.54	0.54	3.51	0.81	8.37
	IRF1	0.27	0	0.54	4.86	0.27	5.94

Genes in bold have not been previously reported to be associated with prognosis of CM patients.

## Data Availability

The raw data are available from the corresponding author upon request.
